# Results of resection of forearm soft tissue sarcoma

**DOI:** 10.1186/s13018-023-04088-7

**Published:** 2023-08-14

**Authors:** Eiji Nakata, Tomohiro Fujiwara, Toshiyuki Kunisada, Ryuichi Nakahara, Haruyoshi Katayama, Takuto Itano, Toshifumi Ozaki

**Affiliations:** Department of Orthopedic Surgery, Okayama University Hospital, 2-5-1, Shikata-Cho, Okayama City, Okayama 700-8558 Japan

**Keywords:** Soft tissue sarcomas, Forearm, Function, Prognosis

## Abstract

**Purpose:**

Soft tissue sarcomas (STS) of the forearm are rare. We aim to assess their oncological and functional outcomes.

**Methods:**

We retrospectively evaluated 34 patients who underwent surgical excision for forearm STS at our institution between 1993 and 2020. We analyzed postoperative Musculoskeletal Tumor Society rating scale (MSTS) and local recurrence-free survival (LRFS), metastasis-free survival, and overall survival (OS) rates. The significance of the following variables was determined: age, sex, histology, tumor size, Fédération Nationale des Centres de Lutte contre le Cancer grade, American Joint Committee on Cancer stage, surgical margin, unplanned excision, metastases upon initial presentation, receipt of chemotherapy, and radiotherapy (RT).

**Results:**

The postoperative median MSTS score was 28. Bone resection or major nerve palsy was the only factor that influenced MSTS scores. The median MSTS scores in patients with or without bone resection or major nerve palsy were 24 and 29, respectively (*P* < 0.001). The 5-year LRFS rates was 87%. Univariate analysis revealed that the histological diagnosis of myxofibrosarcoma was the only factor that influenced LRFS (*P* = 0.047). The 5-year MFS rates was 71%. In univariate analysis, no factors were associated with MFS. The 5-year OS rates was 79%. Age was the only factor that influenced OS (*P* = 0.01).

**Conclusion:**

In the treatment of forearm STS, reconstruction of the skin and tendon can compensate for function, while bone resection and major nerve disturbance cannot. Careful follow-up is important, especially in patients with myxofibrosarcoma, due to its likelihood of local recurrence.

**Supplementary Information:**

The online version contains supplementary material available at 10.1186/s13018-023-04088-7.

## Introduction

Soft tissue sarcomas (STS) of the forearm are rare, accounting for only 3–7% of all sarcomas [1–6]. Several reports have shown the functional and oncological results for the upper or distal upper arm [7–10]. However, limited reports have described the outcomes of STS arising in the forearm [11, 12]. Some authors have reported a high percentage of unplanned excision without preoperative suspicion of malignancy in as much as 25–45% of patients with forearm STS [9, 11]. The association between oncological outcomes and unplanned excision is controversial in STS. Some authors reported higher rates of local recurrence and worse local recurrence-free survival (LRFS) in patients with unplanned excision than in those who underwent planned excision; however, others reported no association between them [13–16].

The goal of forearm STS treatment is to perform limb-sparing surgery with wide resection margins, while preserving the function of the distal upper extremity [4, 5]. However, this becomes challenging because of the complex anatomy and limited tissue volume of the forearm. To obtain optimal oncological and functional outcomes, surgical excision with wide margins followed by reconstruction of the skin, tendons, nerves, vessels, bones, and joints should be performed.

To the best of our knowledge, only three reports have assessed the functional outcomes of forearm STS [9, 11, 12]. However, no study has investigated the factors associated with functional outcomes in forearm STS. The oncological outcome of forearm STS is generally poor, with local recurrence occurring in 7–38% of patients and distant metastases in 13–24% of patients [7, 9, 11, 12]. However, the factors associated with these oncological outcomes have not been fully investigated. Therefore, we analyzed the functional and oncological outcomes of forearm STS.

## Patients and methods

### Study population

We retrospectively evaluated the medical records of 34 patients with forearm STS who underwent surgery at our institution between October 1993 and August 2020 (21 men and 13 women; median range, 64 years [17 to 88]). Patients excluded were those followed up for less than two years after surgery. Those living were followed up for a minimum of 24 months (median range, 89 months [51–297]) (Table 1).

**Table 1:** Patient characteristics

Characteristics	Category	Patients, number
Sex	Male	21
	Female	13
Age, year	Median (range)	64 (17–88)
Tumor size (cm)	Median (range)	5.3 (1.1–14)
Histology	Myxofibrosarcoma	10
	Undifferentiated pleomorphic sarcoma	8
	Undifferentiated sarcoma	3
	Synovial sarcoma	3
	Epithelioid sarcoma	3
	Others	7
Histological grade (FNCLCC)	Grade 1	7
	Grade 2	17
	Grade 3	10
AJCC stage	IA	3
	IB	4
	II	13
	IIIA	7
	IIIB	4
	IV	3

*FNCLCC* Fédération Nationale des Centres de Lutte contre le Cancer, *AJCC* American Joint Committee on Cancer

### Imaging

Computed tomography (CT) or 2-deoxy-2-(18F) fluoro-D-glucose positron emission tomography (FDG-PET) combined with CT (PET/CT) of the chest and abdomen were performed in all patients to determine the presence of distant metastasis upon initial presentation or at follow-up. Magnetic resonance imaging (MRI) was used to evaluate the signal intensity, tumor size, and localization of the tumors. We examined CT images of the chest and abdomen and treated limb looking for a distant metastasis or local recurrence at follow-up; every 4 months in the first 3 years, then twice a year up to the fifth year and once a year thereafter for high-grade sarcomas and every 6 months in the first 3 years, then once a year thereafter for low-grade sarcomas.

### Diagnosis

Histological diagnosis was established by the WHO Classification of Tumors for all patients [17]. The grade was determined using the Fédération Nationale des Centres de Lutte contre le Cancer (FNCLCC) grading system [18]. Myxofibrosarcoma was most diagnosed in ten patients, followed by undifferentiated pleomorphic sarcoma (UPS) in eight patients, and epithelioid sarcoma in three patients each, and others. The median tumor dimension was 5.3 cm (1.1–14). There were grade 1 in 7 patients, grade 2 in 17, and grade 3 in 10. AJCC stages were IA in three patients, IB in four, II in 13, IIIA in seven, IIIB in four, and IV in three. Tumors were superficial in 23 patients and deep in 11 patients. The right side was affected in 18 patients, and the left side was affected in 16 patients, with 19 patients having their dominant hand involved (18 right-sided, one left-sided).

### Surgical treatment

Surgical excision margins were estimated based on the American Joint Committee on Cancer (AJCC) residual tumor classification (R classification); R0 in 31 patients and R1 in 3 patients [19]. Multidisciplinary treatment was performed for tissue reconstruction. Soft tissue coverage was necessary in 25 patients. These were 18 free flaps; anterolateral thigh (ALT) flap in 15 patients, latissimus dorsi (LD) flap in 2, superficial circumflex iliac artery perforator (SCIP) flap in 1, rotation flap from the upper arm in 1, and split thickness skin graft (STSG) in 6. We usually reconstruct flexor and extensor tendon defects of the fingers. These were flexor digitorum profundus (FDP), extensor digitorum (ED), thumb; flexor pollicis longus (FPL) and extensor pollicis longus (EPL), and wrist when they are severely sacrificed. In cases of tendon defects without muscle belly defects, reconstruction is performed with standard tendon autografts (palmaris longus and fascia latae). If the muscle bellies must be sacrificed, then we utilize standard tendon transfer techniques for these cases, including radial nerve palsy, median nerve palsy, or brachial plexus injuries [20, 21]. We performed tendon reconstruction in eight patients (finger in four patients, thumb in one patient, and both in three patients) (Table 2). ED was reconstructed in four patients by transfer to FCR (two patients), extensor carpi radialis longus (one patient), and extensor indicis proprius (one patient). FDP was reconstructed in three patients: autograft of the iliotibial band in two patients and transfer to the flexor digitorum superficialis in one patient. FPL was reconstructed in one patient by transfer to the flexor carpi ulnaris. EPL was reconstructed in three patients by transfer to the palmaris longus. Three patients underwent ulnar resection to achieve adequate margins. Two patients underwent bone resection upon their initial surgery, while the other had it upon recurrence. One patient underwent reconstruction with free vascularized fibular graft (FVFG) for defects of the diaphysis of the ulna; another patient underwent reconstruction of the distal radioulnar joint (DRUJ) to resect the distal ulna using the Sauve-Kapandji method. One patient underwent resection of the diaphysis of the distal ulna, without reconstruction upon recurrence. In nine patients, the radial and ulnar arteries were sacrificed without vascular reconstruction in five and four patients, respectively. In one patient, the ulnar nerve was sacrificed due to tumor adherence, without reconstruction.

**Table 2:** Reconstruction of tendon

Patient	Reconstruction procedure	Recipient	Donor	MSTS score
1	Tendon transfer	EPL	PL	25
		ED	FCR	
	Tenodesis	APL		
2	Tendon transfer	EPL	PL	28
		ED	ECRL	
3	Tendon transfer	EPL	PL	25
	Graft (iliotibial band)	FDP		
4	Tendon transfer	ED (3, 4th), EDM	FCR	30
5	Tendon transfer	ED (4, 5th)	EI	30
6	Graft (iliotibial band)	FDS, FDP (4, 5th)		29
7	Tendon transfer	FDP	FDS	22
8	Tendon transfer	FPL	FCU	29

*MSTS* Musculoskeletal Tumor Society rating Scale, *APL* abductor pollicis longus, *ECRL* extensor carpi radialis longus, *ED* extensor digitorum, *EDM* extensor digiti minimi, *EI* extensor indicis, *EPL* extensor pollicis longus, *FCU* flexor carpi ulnaris, *FCR* flexor carpi radialis, *FDP* flexor digitorum profundus, *FDS* flexor digitorum superficialis, *FPL* flexor pollicis longus, *PL* palmaris longus

### Adjuvant and neoadjuvant therapy

RT was administered to seven patients: one patient with R1 margin and six patients with R0 margin. It was performed preoperatively in one patient and postoperatively in six patients. The radiation absorbed dose was 40–66 Gy with a boost. Chemotherapy was administered to eight patients: one patient as a neoadjuvant, two patients as adjuvant, and five patients used a combination of both.

### Assessment of study outcomes

To investigate the clinical characteristics of the unplanned and planned excision groups, we utilized the following variables: age, sex, FNCLCC grade, AJCC stage, tumor size, depth, and location of the tumor. Functional outcomes of the forearm after surgery were investigated using the MSTS rating scale [22]. We utilized the MSTS score at the last follow-up.

We determined the association of the following variables with the MSTS score: age, unplanned excision, resection of bone/major nerve palsy, the use of free flap, tendon reconstruction, receipt of chemotherapy, and RT.

LRFS was calculated from the date of surgery to the date of local recurrence or to the date of the last follow-up. MFS was calculated from the date of diagnosis to the date of metastasis diagnosis or the last follow-up. OS was calculated from the date of diagnosis to the date of death or the last follow-up visit. Survival rates were estimated using the Kaplan–Meier method. We determined the association of the following variables in terms of survival: age, sex, histology, tumor size, FNCLCC grade, surgical margin, unplanned excision, metastasis upon initial presentation, receipt of chemotherapy, and RT.

The Mann–Whitney U test was used to analyze continuous parameters, while Fisher's exact test was used for categorical parameters. For all analyses, associations were considered significant at a *P* < 0.05, and we used the Bell Curve for Excel (Social Survey Research Information Co., Ltd., Tokyo, Japan).

## Results

### Characteristics of unplanned excision in forearm STS

Fourteen patients (41%) were referred to our institution following an unplanned excision. The median size of tumors in the unplanned excision group was significantly smaller than that of the planned excision group (3.2 cm [range 1.1–9.5] and 6.1 cm [range 2–13.8], respectively [*P* < 0.05]). Subsequently, tumor size, grade, and stage were factors associated with the receipt of unplanned excision (Additional file 1: Table S1).

### Functional outcomes following resection of forearm STS

The median MSTS score for all patients was 28 (range 18–30). Bone resection or major nerve palsy was the only factor influencing the postoperative MSTS score. The median MSTS scores in patients without bone resection or major nerve disturbance were 29 (range 18–30). However, the median MSTS scores in patients with bone resection or major nerve disturbance were 24 (range 18–25), which was significantly worse than that of patients without bone resection (*P* < 0.001). There was no significant difference in MSTS scores according to the reconstruction procedures (the use of flap or tendon reconstruction) (Table 3).

**Table 3:** Risk factors of MSTS

Variable	Category	Median	*p* value
Age, years	< 65	29 (20–30)	0.83
	≥ 65	27 (18–30)	
Unplanned excision	Yes	29 (20–30)	0.40
	No	28 (18–30)	
Resection of bone/nerve palsy	Yes	24 (18–25)	0.001
	No	29 (24–30)	
Free flap	Yes	28 (20–30)	0.64
	No	29 (18–30)	
Tendon reconstruction	Yes	29 (22–30)	0.80
	No	28 (18–30)	
Chemotherapy	Yes	29 (20–30)	0.43
	No	28 (18–30)	
Radiotherapy	Yes	29 (20–30)	0.86
	No	28 (18–30)	

### Postoperative complications

Postoperative complications occurred in six patients. These complications were associated with tendon resection in one patient, flap reconstruction in two patients, and neurological disturbance in three patients. One patient developed finger contractures after tendon resection. Partial necrosis of the flap occurred in two patients; one patient with a pedicled flap healed with conservative treatment, while one with a free flap healed by debridement. Postoperative nerve palsy occurred in three patients. Ulnar nerve palsy was seen in two patients due to the sacrifice of the ulnar nerve in one and surgical procedure in the other. Posterior interosseous nerve palsy was observed in one patient.

### LRFS, MFS, and OS

**Fig. 1:**
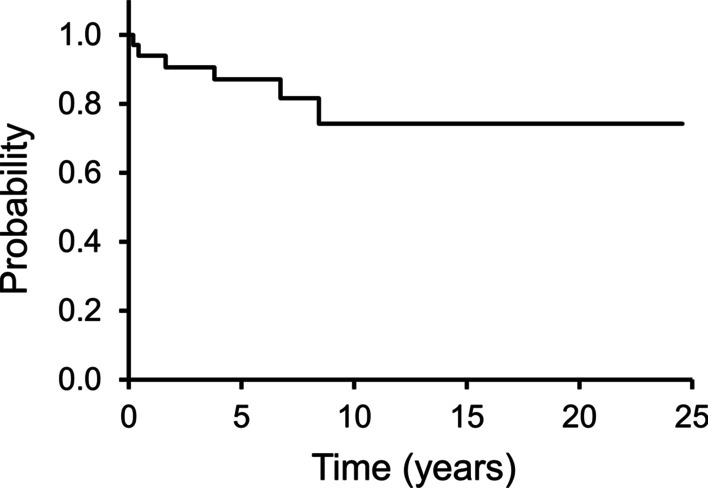
This Kaplan–Meier curve shows local recurrence-free survival (LRFS). The 3- and 5-year LRFS rates are 91% and 87%, respectively

**Fig. 2:**
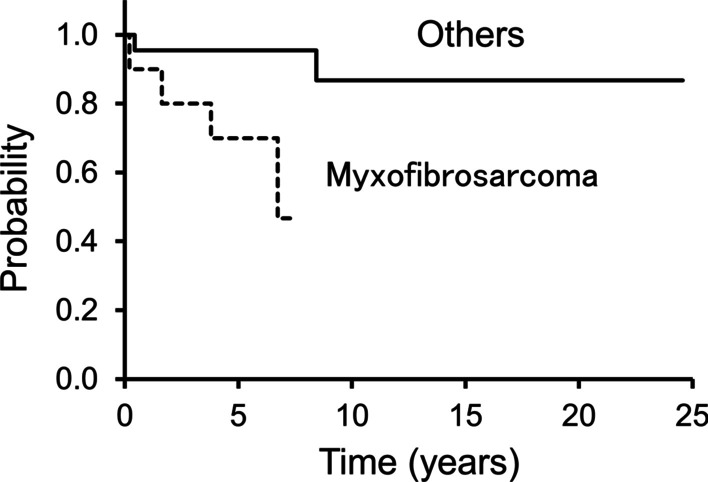
This figure shows local recurrence-free survival (LRFS) in patients with myxofibrosarcoma and others. The 5-year LRFS rates are 70% and 95% in patients with myxofibrosarcoma and others, respectively

Six (18%) had a local recurrence, with a median recurrence period of 27 months (range 3–91 months). The 5-year LRFS rates was 87% (Fig. 1). The histological diagnosis of myxofibrosarcoma was the only factor that influenced LRFS (*P* = 0.047) (Additional file 2: Table S2). Among ten patients with myxofibrosarcoma, four experienced local recurrence, with 5-year LRFS rates being 70% (Fig. 2). On the contrary, 2/22 patients with other tumors, 1 UPS and 1 EMC, showed local recurrence; moreover, the 5-year LRFS rates was 95% (Fig. 2).

**Fig. 3:**
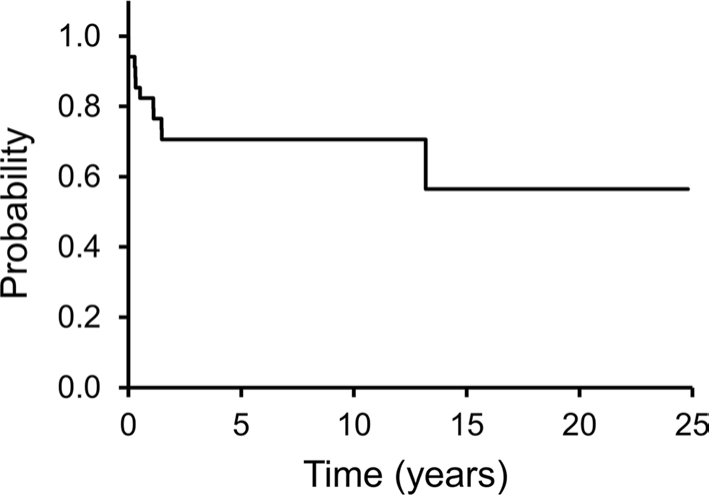
This Kaplan–Meier curve shows metastasis-free survival (MFS). The 3- and 5-year MFS rates are 71% and 71%, respectively

Three patients had distant metastases at presentation; the lungs were involved in one of the patients (UPS), while the others had lymph node involvement (alveolar rhabdomyosarcoma in one patient and epithelioid sarcoma in the other patient). Eight patients had distant metastases during the follow-up period, including six diagnosed with lung metastasis and two with lymph node metastasis. The 5-year MFS rates was 71% (Fig. 3). No factors were associated with MFS (Additional file 3: Table S3).

**Fig. 4:**
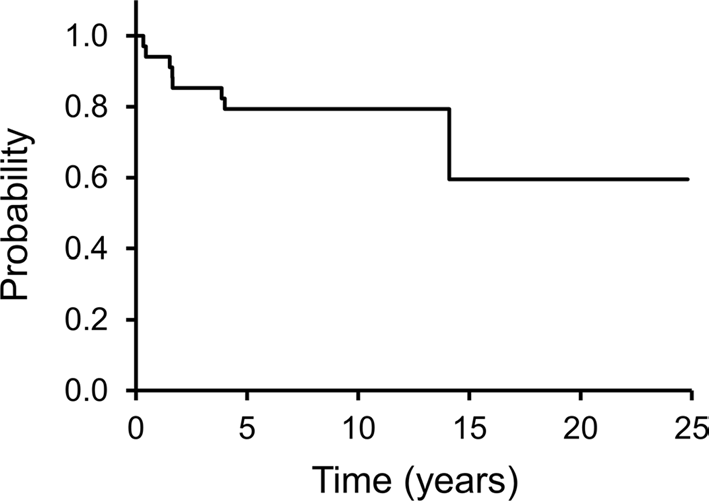
This Kaplan–Meier curve shows overall survival (OS). The 3- and 5-year OS rates are 85% and 79%, respectively

**Fig. 5:**
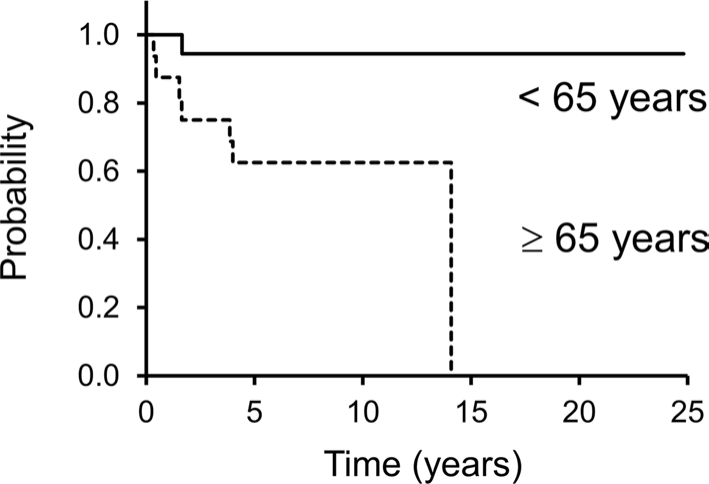
This figure shows overall survival (OS) in patients ≥ 65 years and those with < 65 years. The 5-year OS rates are 63% and 94% in patients ≥ 65 years and < 65 years, respectively

Eight patients were dead by the last follow-up. The 5-year OS rates was 79% (Fig. 4). Age was the only factor that influenced OS (*P* = 0.01) (Additional file 4: Table S4). Among 16 patients aged ≥ 65 years, seven died; the 5-year OS rates was 63% (Fig. 5). Furthermore, one of 18 patients aged < 65 years died; the 5-year OS rates was 94%.

## Discussion

In the treatment of forearm STS, it is often difficult to achieve wide margin due to the vicinity of important vascular and nervous structures [1, 4, 23]. To restore the function after resection of the tumor, multidisciplinary team treatment including orthopaedic oncologist, hand surgeon, and plastic surgeon is important [24].

Few studies have reported the functional outcomes of forearm STS. Muramatsu et al. reported median MSTS scores of 29.5 in eight patients who received microvascular reconstruction [9]. In the study by Bray et al., the functional outcomes were better in patients with forearm STS than in those with STS in the hand and wrist, with a mean TESS of 94 versus 88 [7]. These reports showed the possibility of good to excellent functional outcomes of forearm sarcoma, regardless of the receipt of adjuvant therapy or microvascular reconstruction. Similar to these reports, we found a median MSTS score of 28. In the case of skin defects, which cannot be covered by STSG, local pedicled or perforating flaps can be utilized [25, 26]. Kang et al. reported that the flap reconstruction group had a lower MSTS score and higher wound complication rate but had better local control than those in the primary closure group in patients with STS of the upper extremity [27]. Others found that there was no significant difference in complication rate and functional outcomes between the pedicled and free flap groups in the upper extremity [28, 29]. In this study, we first found equivalent limb function measured by MSTS with or without the use of a free flap. Thus, the free flap is a safe and reliable procedure without impairing forearm function. To the best of our knowledge, only one report has assessed the functional outcomes of forearm sarcoma with tendon reconstruction. Muramatsu et al. reported four cases with defects of the flexor or extensor forearm muscle after tumor resection that received functional neurovascular musculocutaneous flaps to reconstruct finger flexors and extensors [9]. Reinnervation of the transferred muscle was obtained in all cases, and functional outcomes were evaluated as good to excellent, with a median MSTS score of 28. We found equivalent limb function measured by the MSTS in patients with or without tendon reconstruction, suggesting that the function of the forearm can be compensated by tendon reconstruction. We found that bone resection or neurological disturbance led to major loss of function. Preserving the major nerves is extremely important in the forearm, as sacrificing a major nerve leads to a major loss of function. Then, careful preoperative planning and adjuvant treatment may be necessary for preserving the major nerves.

To the best of our knowledge, only one report has assessed survival and its associated risk factors in patients with forearm STS [11]. Baroudi et al. reported a local recurrence rate of 7% and a 5-year LRFS rate of 94% [11]. In this study, the local recurrence rate was 18%, while the 5-year LRFS rate was 87%. The histological diagnosis of myxofibrosarcoma was the only factor that influenced LRFS. Myxofibrosarcoma has a locally infiltrative behavior and is associated with a high local recurrence rate of 24–44% [30–32]. In line with previous studies, a high rate of local recurrence was observed in this study; 4/10 myxofibrosarcoma patients experienced local recurrence, although all of them had achieved R0 margins. The 5-year LRFS rates of myxofibrosarcoma and others were 70% and 95%, respectively (*P* = 0.047). Although wide resection margins were presumed to result in good local control of forearm STS, no relationship between local recurrence and surgical margins was confirmed in this study. The association between surgical margin and local recurrence is controversial. Baroudi et al. also reported no relationship between local recurrence and surgical margins in forearm STS [11]. On the contrary, Heer et al. showed that 4 out of 10 (40%) marginal excisions had local recurrence whereas none had local recurrence in patients with wide excisions [12]. Thus, the utility of surgical margins for local control of forearm STS should be investigated in a larger study.

Baroudi et al. reported a 5-year MFS rate of 74% and that the extra-compartment site was associated with a poor prognosis [11]. In this study, the 5-year MFS rate was 71%. Baroud et al. reported a 5-year OS rate of 81% and described that large (> 4 cm) and soft tissue reconstruction were associated with a poor prognosis [11]. In this study, the 5-year OS rate was 79%, while age (≥ 65 years) was the only factor influencing OS.

This study has several limitations. First, there was a small sample size of only 34 patients. Forearm STS is relatively rare, accounting for about 3–7% of all STS. This limitation is in consistent with previous studies in which relatively small number of patients had been investigated. This limited our ability to identify factors associated with distant metastases. Second, wide resection was not performed in all patients, because some tumors close to major nerves and vessels were excised with R1 margins; this could have influenced survival rates. However, the sample size was likely insufficient to support this. Third, we were unable to compare functional results of those who had reconstructed tendons or skin and those who had not reconstructed them after their removal. Therefore, we compared the forearm function in patients with reconstructed tendons or skin and those who had preserved them. There was no significant difference in MSTS scores according to the reconstruction procedures (the use of flap or tendon reconstruction). Then, we concluded that the function of the forearm can be compensated by tendon reconstruction or flap.

## Conclusion

In conclusion, physicians should be careful in treating forearm tumors when they are suspicious of malignancy. Soft tissue reconstruction using tendons can compensate for function, although bone resection and major nerve disturbances may aggravate the condition. Careful attention during follow-up is important, especially in patients with myxofibrosarcoma, to aid local control.

### Supplementary Information

**Additional file 1: Table S1.** Associated factors of unplanned excision.

**Additional file 2: Table S2.** Risk factors of local recurrence.

**Additional file 3: Table S3.** Risk factors of metastases free survival.

**Additional file 4: Table S4.** Risk factors of overall survival.

## Data Availability

The datasets used and analyzed during the current study are available from the corresponding author on reasonable request.
