# Supplementary material for: Results of resection of forearm soft tissue sarcoma
Source: J Orthop Surg Res. 2023 Aug 14;18:599. doi: 10.1186/s13018-023-04088-7 (PMC10424346; doi:10.1186/s13018-023-04088-7)
Supplement: Supplementary file 1 — Additional file 1: Table S1. Associated factors of unplanned excision. [file 13018_2023_4088_MOESM1_ESM.docx]

**Supplementary table 1.** Associated factors of unplanned excision

FNCLCC; Fédération Nationale des Centres de Lutte contre le Cancer

AJCC; American Joint Committee on Cancer

| Variable | Category | Patients, number | |  |
| --- | --- | --- | --- | --- |
|  |  | Patients with  unplanned excision | Patients without  unplanned excision | p-Value |
| Age, years | < 65 | 9 | 9 | 0.31 |
|  | ≥ 65 | 5 | 11 |  |
|  |  |  |  |  |
| Sex | Male | 7 | 13 | 0.49 |
|  | Female | 7 | 7 |  |
|  |  |  |  |  |
| Tumor size | < 2cm | 4 | 0 | 0.02 |
|  | ≥ 2cm | 10 | 20 |  |
|  |  |  |  |  |
| FNCLCC grade | Grade 1, 2 | 13 | 11 | 0.02 |
|  | Grade 3 | 1 | 9 |  |
|  |  |  |  |  |
| AJCC stage | IA, IB, II | 12 | 8 | 0.01 |
|  | IIIA, IIIB, IV | 2 | 12 |  |
|  |  |  |  |  |
| Depth | Superficial | 12 | 11 | 0.08 |
|  | Deep | 2 | 9 |  |
|  |  |  |  |  |
| Location | Palmor | 8 | 13 | 0.73 |
|  | Dorsal | 6 | 7 |  |
