# Supplementary material for: Results of resection of forearm soft tissue sarcoma
Source: J Orthop Surg Res. 2023 Aug 14;18:599. doi: 10.1186/s13018-023-04088-7 (PMC10424346; doi:10.1186/s13018-023-04088-7)
Supplement: Supplementary file 2 — Additional file 2: Table S2. Risk factors of local recurrence. [file 13018_2023_4088_MOESM2_ESM.docx]

**Supplementary table 2.** Risk factors of local recurrence

| Variable | Category | Patients, number | |  |
| --- | --- | --- | --- | --- |
|  |  | Patients with  local recurrence | Patients without  local recurrence | p-Value |
| Age, years | < 65 | 4 | 14 | 0.66 |
|  | ≥ 65 | 2 | 14 |  |
|  |  |  |  |  |
| Sex | Male | 3 | 17 | 0.67 |
|  | Female | 3 | 11 |  |
|  |  |  |  |  |
| Histology | Myxofibrosarcoma | 4 | 6 | 0.047 |
|  | Others | 2 | 22 |  |
|  |  |  |  |  |
| Tumor size | < 2cm | 1 | 3 | 0.96 |
|  | ≥ 2cm | 5 | 25 |  |
|  |  |  |  |  |
| FNCLCC grade | Grade 1 | 3 | 4 | 0.08 |
|  | Grade 2,3 | 3 | 24 |  |
|  |  |  |  |  |
| Margin | R0 | 4 | 27 | 0.07 |
|  | R1 | 2 | 1 |  |
|  |  |  |  |  |
| Unplanned excision | Yes | 4 | 10 | 0.51 |
|  | No | 2 | 18 |  |
|  |  |  |  |  |
| Metastases upon initial presentation | Yes | 2 | 1 | 0.30 |
|  | No | 4 | 27 |  |
|  |  |  |  |  |
| Chemotherapy | Yes | 0 | 8 | 0.30 |
|  | No | 6 | 20 |  |
|  |  |  |  |  |
| Radiotherapy | Yes | 0 | 7 | 0.31 |
|  | No | 6 | 21 |  |

FNCLCC; Fédération Nationale des Centres de Lutte contre le Cancer
