# Supplementary material for: Results of resection of forearm soft tissue sarcoma
Source: J Orthop Surg Res. 2023 Aug 14;18:599. doi: 10.1186/s13018-023-04088-7 (PMC10424346; doi:10.1186/s13018-023-04088-7)
Supplement: Supplementary file 3 — Additional file 3: Table S3. Risk factors of metastases free survival. [file 13018_2023_4088_MOESM3_ESM.docx]

**Supplementary table 3.** Risk factors of distant metastases

| Variable | Category | Patients, number | |  |
| --- | --- | --- | --- | --- |
|  |  | Patients with  metastases | Patients without  metastases | p-Value |
| Age, years | < 65 | 4 | 13 | 1.00 |
|  | ≥ 65 | 4 | 10 |  |
|  |  |  |  |  |
| Sex | Male | 5 | 12 | 0.70 |
|  | Female | 3 | 11 |  |
|  |  |  |  |  |
| Histology | Myxofibrosarcoma | 2 | 8 | 0.69 |
|  | Others | 6 | 15 |  |
|  |  |  |  |  |
| Tumor size | < 2cm | 0 | 4 | 0.55 |
|  | ≥ 2cm | 8 | 19 |  |
|  |  |  |  |  |
| FNCLCC grade | Grade 1 | 2 | 5 | 1.00 |
|  | Grade 2,3 | 6 | 18 |  |
|  |  |  |  |  |
| Margin | R0 | 7 | 21 | 1.00 |
|  | R1 | 1 | 2 |  |
|  |  |  |  |  |
| Unplanned excision | Yes | 3 | 11 | 0.70 |
|  | No | 5 | 12 |  |
|  |  |  |  |  |
| Chemotherapy | Yes | 1 | 5 | 1.00 |
|  | No | 7 | 18 |  |
|  |  |  |  |  |
| Radiotherapy | Yes | 2 | 4 | 0.63 |
|  | No | 6 | 19 |  |

FNCLCC; Fédération Nationale des Centres de Lutte contre le Cancer
