# Supplementary material for: Results of resection of forearm soft tissue sarcoma
Source: J Orthop Surg Res. 2023 Aug 14;18:599. doi: 10.1186/s13018-023-04088-7 (PMC10424346; doi:10.1186/s13018-023-04088-7)
Supplement: Supplementary file 4 — Additional file 4: Table S4. Risk factors of overall survival. [file 13018_2023_4088_MOESM4_ESM.docx]

**Supplementary table 4.** Risk factors of overall survival

| Variable | Category | Patients, number | |  |
| --- | --- | --- | --- | --- |
|  |  | Patients with death | Patients without death | p-Value |
| Age, years | < 65 | 1 | 17 | 0.01 |
|  | ≥ 65 | 7 | 9 |  |
|  |  |  |  |  |
| Sex | Male | 6 | 14 | 0.42 |
|  | Female | 2 | 12 |  |
|  |  |  |  |  |
| Histology | Myxofibrosarcoma | 1 | 9 | 0.39 |
|  | Others | 7 | 17 |  |
|  |  |  |  |  |
| Tumor size | < 2cm | 0 | 4 | 0.55 |
|  | ≥ 2cm | 8 | 22 |  |
|  |  |  |  |  |
| FNCLCC grade | Grade 1 | 1 | 6 | 1.00 |
|  | Grade 2,3 | 7 | 20 |  |
|  |  |  |  |  |
| Margin | R0 | 6 | 25 | 0.13 |
|  | R1 | 2 | 1 |  |
|  |  |  |  |  |
| Unplanned excision | Yes | 1 | 13 | 0.30 |
|  | No | 7 | 13 |  |
|  |  |  |  |  |
| Metastases upon initial presentation | Yes | 2 | 1 | 0.13 |
|  | No | 6 | 25 |  |
|  |  |  |  |  |
| Chemotherapy | Yes | 1 | 7 | 0.20 |
|  | No | 7 | 19 |  |
|  |  |  |  |  |
| Radiotherapy | Yes | 3 | 4 | 0.51 |
|  | No | 5 | 22 |  |

FNCLCC; Fédération Nationale des Centres de Lutte contre le Cancer
